# Maize Milling By-Products: From Food Wastes to Functional Ingredients Through Lactic Acid Bacteria Fermentation

**DOI:** 10.3389/fmicb.2019.00561

**Published:** 2019-03-19

**Authors:** Erica Pontonio, Cinzia Dingeo, Marco Gobbetti, Carlo Giuseppe Rizzello

**Affiliations:** ^1^Department of Soil, Plant and Food Sciences, University of Bari Aldo Moro, Bari, Italy; ^2^Faculty of Science and Technology, Free University of Bozen-Bolzano, Bolzano, Italy

**Keywords:** maize, milling by-products, lactic acid fermentation, high-fiber, nutritional profile

## Abstract

Although recognized as important sources of functional compounds, milling by-products are often removed from the cereal kernel prior milling process. Indeed, the high presence of fiber in bran and the co-presence of lipids and lipase in germ are often considered as downsides for breadmaking. In this work, *Lactobacillus plantarum* T6B10 and *Weissella confusa* BAN8 were used as selected starters to ferment maize milling by-products mixtures made with heat-treated or raw germ and bran. The effects on the biochemical and nutritional features as well as the stability of the milling by-products were investigated. Lactic acid bacteria metabolisms improved the free amino acids and peptides concentrations and the antioxidant activity and caused a relevant phytic acid degradation. Moreover, fermentation allowed a marked decrease of the lipase activity, stabilizing the matrix by preventing oxidative processes. The use of fermented by-products as ingredients improved the nutritional, textural and sensory properties of wheat bread. Fortified breads (containing 25% of fermented by-products) were characterized by a concentration in dietary fiber and proteins of *ca*. 11 and 13% of dry matter, respectively. Compared to the use of the unfermented ones, the addition of pre-fermented by-products to bread caused a significant increase in protein digestibility (up to 60%), and a relevant decrease of the starch hydrolysis index (*ca*. 13%). According to the results, this study demonstrates the potential of fermentation to convert maize bran and germ, commonly considered food wastes, into nutritive improvers, meeting nutritional and sensory requests of modern consumers.

## Introduction

The World Health Organization (WHO) stated, in 2016, that more than 1.9 billion adults were overweight and more than 600 million obese (WHO, 2018), probably due to the radical changes of the dietary habits recorded through the past decades (Fernstrand et al., 2017). The average daily intake of fiber in many populations is still lower than those recommended (Stephen et al., 2017), although the consumers are already aware of the advantages of a healthy diet rich in dietary fiber. Indeed, numerous physiological effects have been highlighted, i.e., the prevention of coronary heart disease, type 2 diabetes, colorectal and other types of cancers (Kaczmarczyk et al., 2012) as well as it seems to be inversely associated with body weight due to the suppressing effect on the energy intake through increasing satiation (Howarth et al., 2001). Fortification of a staple food, i.e., bread, represents a promising way to increase fiber intake. However, it is needed to be able to produce healthy, fiber-rich bakery products with an appealing texture and taste. Optimal diet rich in fiber refers at the same time to the right amount and a suitable balance between them. The production of multi-grain products makes it possible, providing more variety in breads and increasing the diversity in fermentable soluble fiber (Lopez et al., 2001). The need for food companies to produce dietary fibers-enriched as well as low-calorific foods to meet the consumers requirements, is leading to investments in allocating resources in innovative foods developments. In this scenario, several scientific researches have been carried out aiming at substituting wheat flour in bread formulations (Sivam et al., 2010; Preedy et al., 2011; Rosell et al., 2016) and toward sustainable solutions, the fortification with milling by-products has largely been proposed (Katina et al., 2012; Rizzello et al., 2012; Singh et al., 2012; Pontonio et al., 2017). Bran, the outer layers of cereal grains, is rich in dietary fiber as well as other bioactive compounds and germ is usually characterized by a high nutritional value (Katina et al., 2012). However, despite to the positive effects on health, the presence of high content of fiber in bran and lipase and lipoxygenase activities in germ may negatively affect the baked goods quality. Indeed, use of native bran in wheat baking is a technological challenge because of the detrimental effect of bran on the gluten network and subsequent textural attributes of bread (Noort et al., 2010) other than negative influence on the taste of the products. While, the lipase and lipoxygenase activities determine the poor and unstable sensory properties of baked goods made of wheat flour containing the germ (Paradiso et al., 2008). However, lipases are sometimes used in bakery industry as emulsifiers to increase the bread volume, soften the crumb as well as retarding bread staling (Frauenlob et al., 2018). Thermal treatments can be used to inactivate the enzymes, however, collateral negative effects on the bioactive compounds, i.e., destruction of essential fatty acids and vitamins are often highlighted (Sjovall et al., 2000). Therefore, a careful selection of process to pre-treat milling by-products need to be done. Lactic acid bacteria (LAB) have already been proposed as a promising tool to overcome the sensory, structural, functional and nutritional drawbacks related to their use as ingredients in bread-making (Katina et al., 2012; Rizzello et al., 2012; Pontonio et al., 2017).

Maize (*Zea mays*) is a domesticated grass that originated in what is now Mexico. Worldwide, 60–70% of maize production is used domestically as livestock feed, and the remaining 30–40% is used for production of items for human consumption (Pranjal et al., 2017). Nevertheless, due to the processing wastes and the preparation of non-food products, the use of these cereals is lower than what estimated (Ranum et al., 2014). Milling by-products (bran and germ), which contain most of the bioactive compounds, are often removed from the kernel prior processing thus causing a loss of nutritional quality (Ranum et al., 2014).

In this study, selected LAB were used to ferment raw- and heat-treated milling by-products from maize, investigating the effects of the fermentation on their stabilization biochemical and nutritional properties. Based on the above considerations and aiming at increasing the fiber content of wheat bread valorizing food processing wastes, fermented and unfermented mixtures of maize germ and bran were used as ingredients to fortify wheat breads, evaluating biochemical and nutritional characteristics, structural properties and sensory profiles.

## Materials and Methods

### Bacterial Strains, Growth Conditions and Starter Selection

Aiming at investigating a wide microbial diversity, starters were selected among 100 LAB strains (Supplementary Table S1) previously isolated from matrices with different chemical composition and sharing either most of the functional compounds and anti-nutritional factors with maize milling by-products. In detail, LAB strains belonging to the Culture Collection of the Department of Soil, Plant and Food Science (University of Bari, Italy) were previously isolated from raw or spontaneously fermented wheat, quinoa, hemp, hop, and wheat germ (Rizzello et al., 2010, 2016; Nionelli et al., 2014, 2018a,b; Pontonio et al., 2015; Mamhoud et al., 2016) (Supplementary Table S1). Strains were routinely cultivated on modified de Man, Rogosa and Sharpe medium (mMRS, maltose and fresh yeast extract were added to MRS at 1 and 5%, respectively, and the final pH was 5.6) until the late exponential phase of growth was reached (*ca*. 8 h), as previously determined by the analysis of the kinetics of growth (Rizzello et al., 2010, 2016; Nionelli et al., 2014, 2018a,b; Pontonio et al., 2015; Mamhoud et al., 2016).

Aiming at selecting strains to be used as mixed starter for maize milling by-products fermentation, the pro-technological and functional features of LAB were evaluated when singly inoculated in their own isolation matrix (wheat, quinoa, hemp and hop flours and wheat germ). Cells were harvested by centrifugation (10,000 ×*g*, 10 min, 4°C), washed twice in 50 mM phosphate buffer, pH 7.0, and re-suspended in tap water. The DY (dough yield, dough weight × 100/flour weight) was 200 and the initial cell density of each LAB was *ca*. 7.0 log10 cfu/g. Fermentation was carried out in triplicate at 30°C for 24 h. After fermentation, samples were stored at 4°C and analyzed within 2 h. Non-inoculated doughs were used as controls. Proteolytic by means of total free amino acids (TFAA), phytase and radical scavenging (in the methanolic extract) activities were considered as functional features, however, kinetics of growth and acidification were considered as pro-technological traits.

Kinetics of growth and acidification were determined and modeled in agreement with the Gompertz equation, as modified by Zwietering et al. (1990): *y* = *k* + *A* exp{- exp[(μmax or Vmax e/A)(λ-t) + 1]}; where y is the growth expressed as log10 cfu/g/h or the acidification rate expressed as dpH/dt (units of pH/h) at the time *t*; *k* is the initial level of the dependent variable to be modeled (log10 cfu/g or pH units); A is the cell density or pH (units) variation (between inoculation and the stationary phase); μmax or Vmax is the maximum growth rate expressed as Δ log10 cfu/g/h or the maximum acidification rate expressed as dpH/h, respectively; λ is the length of the lag phase measured in hours. The experimental data were modeled by the non-linear regression procedure of the Statistica 12.0 software (Statsoft, Tulsa, OK, United States). The values of pH of doughs were determined by a M.507 pHmeter (Crimson, Milan, Italy) equipped with a food penetration probe.

Water/salt-soluble extracts (WSE) from doughs were prepared according to the method originally described by Osborne (1907) and modified by Weiss et al. (1993). Briefly, 5g of sample were suspended in 10 ml of 50 mM Tris–HCl (pH 8.8), incubated at 4°C for 1 h under stirring conditions (150 rpm) and centrifuged at 12,000 × *g* for 20 min. The supernatant was used for the determination of TFAA concentration and phytase activity. TFAA were analyzed by a Biochrom 30 series Amino Acid Analyzer (Biochrom Ltd., Cambridge Science Park, United Kingdom) with a Na-cation-exchange column (20 by 0.46 cm internal diameter), as described by Rizzello et al. (2010). Phytase activity was determined by monitoring the rate of hydrolysis of *p*-nitrophenyl phosphate (p-NPP) (Sigma, 104-0). The assay mixture contained 200 μL of 1.5 mM *p*-NPP (final concentration) in 0.2 M Na-acetate, pH 5.2, and 400 μL of WSE. The mixture was incubated at 45°C and the reaction was stopped by adding 600 μL of 0.1 M NaOH. The *p*-nitrophenol released was determined by measuring the absorbance at 405 nm (Rizzello et al., 2010). One unit (U) of activity was defined as the amount of enzyme required to liberate 1 μmol/min of *p*-nitrophenol under the assay conditions. The radical scavenging activity was determined on the ME methanolic extract (ME) of doughs. Three grams of each sample were mixed with 30 ml of 80% (vol/vol) methanol to get ME. The mixture was purged with nitrogen stream for 30 min, under stirring condition, and centrifuged at 4,600 ×*g* for 20 min. The supernatants (MEs) were transferred into test tubes, purged with nitrogen stream and stored at *ca*. 4°C before analysis. The radical DPPH^.^ was used for determining the free radical scavenging activity (Rizzello et al., 2010). The synthetic antioxidant butylated hydroxytoluene (BHT) was included in the analysis as the reference (75 ppm). The reaction was monitored by reading the absorbance at 517 nm.

Based on the results collected under the above conditions, the two best performing strains, *Lactobacillus plantarum* T6B10 and *Weissella confusa* BAN8, were selected and used as a mixed starter for sourdough fermentation of maize milling by-products.

### Microbiological and Chemical Analysis of Milling By-Products

Commercial samples of maize milling by-products, certified for mycotoxins levels (aflatoxins, zearalenone, deoxynivalenol, ochratoxin A, and fumonisin) under the thresholds defined by Reg. UE 1881/2006, Reg. UE 1126/2007, and Reg. UE 165/2010, were supplied by Molino Favero (Padova, Italy). Raw germ (RG), germ stabilized by heat-treatment (TG) and bran (B) were used in this study. The heat-treatment was carried out at *ca*. 200°C until the product temperature of 110°C was achieved. Temperature was monitored during treatment trough an RTD temperature probe (Jumo Food temp insertion RTD 902350, Sesto San Giovanni, Italy). Proximal analysis of the milling by-products prior doughs preparation was carried out.

Protein (total nitrogen × 5.7), lipids, moisture, total dietary fiber and ash of RG, TG and B were determined according to Approved Methods 46-11A, 30-10.01, 44-15A, 32-05.01, and 08-01.01 of the American Association of Cereal Chemists (American Association of Cereal Chemists [AACC], 2010). Available carbohydrates were calculated as the difference [100 - (proteins + lipids + ash + total dietary fiber)]. Proteins, lipids, carbohydrates, total dietary fiber and ash were expressed as % of dry matter (d.m.).

Microbiological analyses were carried out on milling by-products as specified below. In details, 10 g of RG, TG and B were suspended in 90 ml of sterile sodium chloride (0.9%, wt/vol) solution and homogenized in a Stomacher lab blender for 2 min at room temperature. Mesophilic presumptive LAB were determined on mMRS at 30°C for 48–72 h, under anaerobiosis. Yeasts were plated on Sabouraud Dextrose Agar (SDA, Oxoid, Basingstoke, Hampshire, United Kingdom), supplemented with chloramphenicol (0.1 g/l) at 25°C for 48h. Molds were enumerated on Potato Dextrose Agar (PDA, Oxoid) at 25°C for 48 h. Total *Enterobacteria* were determined on Violet Red Bile Glucose Agar (VRBGA, Oxoid) at 37°C for 24 h and total mesophilic bacteria were determined on Plate Count Agar (PCA, Oxoid) at 30°C for 48 h.

### Milling By-Products Fermentation

Doughs (100 g) consisting of milling by-products mixtures and water (1:1) were obtained by an IM 58 high-speed mixer (Mecnosud, Flumeri, Italy). DY was 200. Mixtures of RG and B (ratio 2:1, FMBP_RG_) or TG and B (ratio 2:1, FMBP_TG_) were used. Doughs were inoculated with *L*. *plantarum* T6B10 and *W*. *confusa* BAN8 each at the cell density of *ca*. 7 log10 cfu/g of dough. Fermentations were carried out in triplicate at 30°C for 24 h. After fermentation, samples were stored at 4°C and analyzed within 2 h. Non-fermented doughs (MBP_RG_ and MBP_TG_) were used as controls.

### Microbiological, Biochemical and Nutritional Characterization of Fermented Milling By-Products

Lactic acid bacteria and pH values of MBP_RG_, MBP_TG_, FMBP_RG_, and FMB_TG_ were determined as reported above. Ten grams of MBP_RG_, MBP_TG_, FMBP_RG_, and FMB_TG_ were homogenized with 90 ml of distilled water for the determination of total titratable acidity (TTA). TTA is expressed as the amount (ml) of 0.1 M NaOH to reach pH of 8.3. WSE from fermented and un-fermented doughs were used for the determination of organic acids, peptides, TFAA concentrations and radical scavenging activity. Organic acids were determined by High Performance Liquid Chromatography (HPLC), using an ÄKTA Purifier system (GE Healthcare, Buckinghamshire, United Kingdom) equipped with an Aminex HPX-87H column (ion exclusion, Bio-Rad, Richmond, CA, United States), and an UV detector operating at 210 nm. Elution was at 60°C, with a flow rate of 0.6 ml/min, using H_2_SO_4_ 10 mM as mobile phase (Rizzello et al., 2010). The quotient of fermentation (QF) was determined as the molar ratio between lactic and acetic acids. TFAA were analyzed as reported above. For the peptides analysis, WSE were treated with trifluoroacetic acid (0.05% wt/vol) and subject to dialysis (cut-off 500 Da) to remove proteins and FAA, respectively. Then, peptides concentration was determined by the *o*-phtaldialdehyde (OPA) method as described by Church et al. (1983). All analyses were carried out in triplicate.

Tributyrin was used as the substrate to determine the lipase activity of the MBP_RG_, MBP_TG_, FMBP_RG_, and FMBP_TG_ extract by agar diffusion assay (Lawrence et al., 1967). Agar plates contained 1% (wt/vol) of triglyceride, 0.02% (wt/vol) sodium azide, and 50 mM phosphate buffer, pH 8.0. As reported by Lin et al. (1983), this value of pH was the optimum for maize germ endogenous lipase activity. Activity was expressed as the minimum dilution of the enzyme preparation that failed to give a detectable zone of hydrolysis after 24 h of incubation at 30°C.

Phytic acid concentration was measured using K-PHYT 05/07 kit assay (Megazyme Intl., Ireland), following the manufacturer’s instructions. Total phenols and radical scavenging activity were determined on the ME of MBP_RG_, MBP_TG_, FMBP_RG_, and FMBP_TG_. The concentration was determined as described by Slinkard and Singleton (1977) and expressed as gallic acid equivalent. The radical scavenging activity was determined as reported above.

### Breadmaking

Experimental breads (DY, 180) were manufactured at the pilot plant of the Department of Soil, Plant and Food Science of the University of Bari (Italy), according to the two-stage protocol commonly used for sourdough breadmaking (Rizzello et al., 2016). MBP_RG_ and MBP_TG_ were fermented at 30°C for 24 h with the mixed starters as described before (step I); then, FMBP_RG_ and FMBP_TG_ were mixed with wheat flour, water, and baker’s yeast at 60 ×*g* for 5 min with an IM 58 high-speed mixer (Mecnosud, Flumeri, Italy) and incubated for 1.5 h at 30°C (step II). The characteristics of the flour (*Triticum aestivum*, cv Appulo) used were the following: moisture, 14.2%; protein (N × 5.70), 11.5% of d.m.; fat, 1.6% of d.m.; ash, 0.6% of d.m. and total soluble carbohydrates, 1.5% of d.m. In detail, MBP_RG_ and MBP_TG_ and FMBP_RG_ and FMBP_TG_ were used at the percentage of 12.5 and 25% (wt/wt), respectively, of the total dough weight (Nionelli et al., 2014). At the end of step II, doughs (300 g) were baked at 220°C for 50 min (Combo 3, Zucchelli, Verona, Italy), obtaining breads fortified with raw and fermented milling by-products (MBP_RG_-B/MBP_TG_-B and FMBP_RG_-B/FMBP_TG_-B, respectively). A baker’s yeast wheat bread (WB) was manufactured without the addition of milling by-products (DY, 180) and used as the control. Baker’s yeast was added at the percentage of 1.5% (wt/wt), corresponding to a final cell density of *ca*. 9 log10 cfu/g in all the doughs only for the step II. Salt was not used. All breads were cooled for a period of 2 h on cooling racks at room temperature prior analysis.

The Texture Profile Analysis (TPA) of bread was carried out by means of a Universal Testing machine (model 3344, Instron, Norwood, MA, United States), equipped with 3.6 cm diameter cylindrical probe, 1000 N load cell. The chromaticity co-ordinates of the bread crust (obtained by a Minolta CR-10 camera) were also reported in the form of a color difference, dE^∗^ab, as follows:

$$dE*ab=\sqrt{{\left(\text{d}L\right)}^{2}+{\left(\text{d}a\right)}^{2}+{\left(\text{d}b\right)}^{2}}$$

where dL, da, and db are the differences for *L*, *a*, and *b* values between sample and reference a white ceramic plate having *L* = 67.04, *a* = 2.44, and *b* = 18.28.

The values of pH and TTA, concentration of organic acids, TFAA, total phenols and phytic acid and radical scavenging activity were determined as reported above. The specific volume and moisture content of breads were measured determined according to the approved methods AACC 10-05.01 and 44-15.02, respectively (American Association of Cereal Chemists [AACC], 2010). Water activity (*a*_w_) was determined at 25°C by the Aqualab Dew Point 4TE water activity meter (Decagon Devices Inc., United States). Fermentations were carried out in triplicate and each bread was analyzed twice.

### Nutritional Characterization of Breads

The *in vitro* protein digestibility (IVPD) of breads was determined by the method proposed by Akeson and Stahmann (1964) with some modifications (Rizzello et al., 2014). Samples were subjected to a sequential enzyme treatment mimicking the *in vivo* digestion in the gastro intestinal tract and IVPD was expressed as the percentage of the total protein which was solubilized after enzyme hydrolysis. The concentration of protein of digested and non-digested fractions was determined by the Bradford method (Bradford, 1976). The analysis of starch hydrolysis was carried out on breads. The procedure mimicked the *in vivo* digestion of starch (De Angelis et al., 2009). Aliquots of breads, containing 1 g of starch, were undergo to enzymatic process and the released glucose content was measured with D-Fructose/D-Glucose Assay Kit (Megazyme). The degree of starch digestion was expressed as the percentage of potentially available starch hydrolyzed after 180 min. Wheat flour bread (WB) leavened with baker’s yeast was used as the control to estimate the hydrolysis index (HI = 100). The predicted GI was calculated using the equation: GI = 0.549 × HI + 39.71 (Capriles and Areas, 2013).

### Sensory Analysis

Sensory analysis of breads was carried out by 10 trained panelists (5 male and 5 females, mean age: 35 years, range: 18–54 years), according to the method described by Haglund et al. (1998). After a roundtable discussion about the attributes, 13 were selected as the most frequently recognized by all the members of the panel. These were included in a score sheet for the quantitative evaluation with a scale from 0 to 10, with 10 the highest score. Elasticity of crumb, softness of crumb, crust and crumb color were considered visual/kinesthetic attributes Taste was evaluated as nuts, sourness, bitterness and toasted, while for aroma was evaluated the intensity, the rancid and aromatic characteristics of mixed nuts (Georgsson, 2015). Besides, the typical aroma of a fermented dough was evaluated. According to the IFST Guidelines for Ethical and Professional Practices for the Sensory Analysis of Foods, assessors gave informed consent to tests and could withdraw from the panel at any time, without penalty or having to give a reason.

### Statistical Analysis

Data were subjected to one-way ANOVA; pair-comparison of treatment means was achieved by Tukey’s procedure at *P* < 0.05, using the statistical software, Statistica 12.5 (TIBCO Software Inc., Palo Alto, CA, United States) for Windows. Principal Components analysis was performed through Xlstat 2014 (Addinsoft, New York, NY, United States).

## Results

### Starters Selection for Lactic Acid Fermentation

**FIGURE 1:**
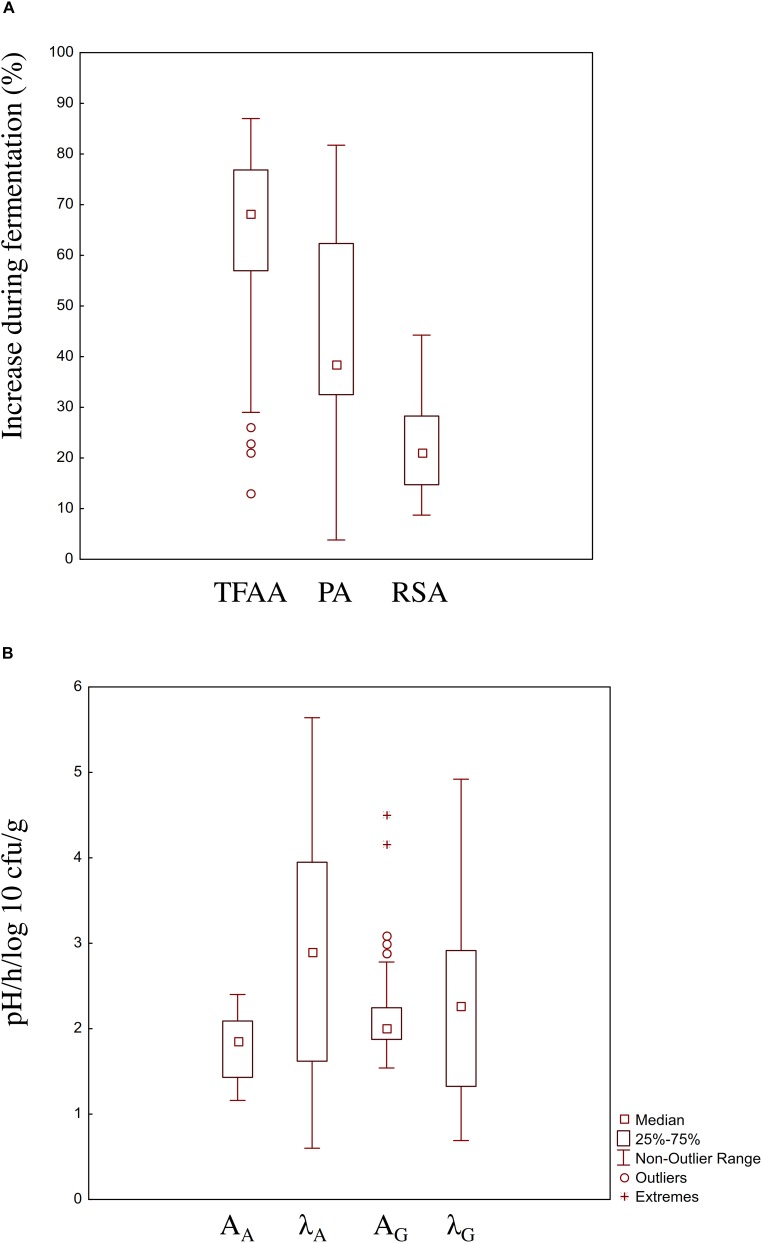
Boxplot showing the functional **(A)** and pro-technological **(B)** characterization of 100 strains of lactic acid bacteria belonging to the species *Lactobacillus brevis*, *Lactobacillus curvatus*, *Lactobacillus helveticus*, *Lactobacillus farciminis*, *Lactobacillus nantensis*, *Lactobacillus plantarum*, *Lactobacillus rossiae*, *Lactococcus lactis*, *Pediococcus acidilactici*, *Pediococcus pentosaceus*, *Weissella cibaria*, *Weissella confusa*, *Leuconostoc citreum*, and *Leuconostoc mesenteroides* of the Culture Collection of the Department of Soil, Plant and Food Science of the University of Bari, Italy and isolated from raw or spontaneously fermented wheat, hemp, hop, quinoa, wheat germ and bran. The increase (%) of TFAA concentration, phytase (PA) and radical scavenging (RSA) activities in wheat, hemp, hop, quinoa, wheat germ and bran (DY 200) singly inoculated with the strains and fermented for 24 h at 30°C, compared to a not inoculated dough incubated in the same conditions were considered as functional features. Panel B displays the boxplot of the acidification (*A*_A_, pH; λ_A_, h) and growth (*A*_G_, log 10 cfu/g; λ_G_, h) kinetics parameters of the strains in the above-mentioned conditions, respectively. The top and the bottom of the box represent the 75th and 25th percentile of the data, respectively. The top and the bottom of the bars represent the 5th and the 95th percentile of the data, respectively.

Lactic acid bacteria strains were singly used to ferment wheat, quinoa, hemp and hop flours and wheat germ at 30°C for 24 h. To allow the comparison between results from different food matrices, the increase (%) of TFAA concentration and phytase and radical scavenging activities, as compared to the corresponding non-inoculated doughs, were considered (Figure 1A). Increases of TFAA were in the range 13–87%, being the highest for *L*. *plantarum* T6B10 and the lowest for *Lactobacillus farciminis* S3N2. Similarly, wide increase of the phytase activity was found among the LAB strains, with highest and lowest values reached when *L*. *plantarum* T6B10 (81.7%) and *Lactococcus lactis* LVS 26 (3.8%) were used, respectively. Highest value of radical scavenging activity (44.3%) were found when *L*. *plantarum* LIN 2 was used to ferment wheat flour (Figure 1A). According to the pro-technological features, *W*. *confusa* BAN8 showed highest cell density increase (A_G_, 2.4 log10 cfu/g). Moreover, both *L*. *plantarum* T6B10 and *W*. *confusa* BAN8 fell into the 75 and 25% percentile of the *A*_A_ and λ_A_ and λ_G_, respectively (Figure 1B).

Based on the above results, *L*. *plantarum* T6B10 and *W*. *confusa* BAN were selected and used as mixed starter to ferment maize milling by-products.

### Milling By-Products Characterization

The proximal composition and microbiological characterization of RG, TG and B used in this study are reported in Table 1. The heat-treatment led to a TG having moisture four times lower than RG. Although B was also subjected to heat-treatment, its moisture was 10.6 ± 0.9%. As expected, RG and TG contained high level of fat (up to *ca*. 33% of d.m.) and B was characterized by the highest concentration of carbohydrates and especially total dietary fiber (up to *ca*. 50%). Probably due to the heat treatment, any of the microbial groups investigated were detectable in 1 g TG, however, total mesophilic bacteria and molds were detected in B at cell density ≤ 2 log10 cfu/g (Table 1).

**Table 1:** Proximal composition and microbiological characterization of the maize raw (RG) and heat-treated (TG) germ and bran (B).

	RG	TG	B
**Proximal composition^∗^**			
Moisture (%)	7.9 ± 0.3^b^	2.3 ± 0.2^a^	10.6 ± 0.9^c^
Protein (%)	21.9 ± 0.2^b^	19.8 ± 0.2^a^	18.4 ± 0.6^a^
Fat (%)	32.3 ± 0.3^b^	33.5 ± 0.8^c^	3.2 ± 0.1^a^
Available carbohydrates (%)	9.5 ± 0.9^b^	5.7 ± 0.9^a^	23.3 ± 0.9^c^
Total dietary fibers (%)	32.7 ± 0.8^a^	32.7 ± 0.4^a^	50.0 ± 0.8^b^
Ash (%)	8.2 ± 0.4^b^	8.3 ± 0.6^b^	5.0 ± 0.2^a^
**Microbiological characterization**			
Mesophilic aerobic bacteria (log10 cfu/g)	5.3 ± 0.7^c^	<10 cfu/1g^a^	2.0 ± 0.3^b^
Yeast (log10 cfu/g)	<10 cfu/1g^a^	<10 cfu/1g^a^	< 10 cfu/1g^a^
Molds (log10 cfu/g)	4.3 ± 0.6^c^	<10 cfu/1g^a^	1.5 ± 0.2^b^
LAB (log10 cfu/g)	3.5 ± 0.5^b^	<10 cfu/1g^a^	<10 cfu/1g^a^
*Enterobacteriaceae* (log10 cfu/g)	2.0 ± 0.3^b^	<10 cfu/1g^a^	<10 cfu/1g^a^

*The data are the means of three independent experiments ± standard deviations (n = 3); ^∗^Data of protein, fat, carbohydrates, total fiber and ash are expressed on dry weight; LAB, Lactic acid bacteria; ^a-c^Values in the same row with different superscript letters differ significantly (p < 0.05).*

### Fermented Milling By-Products Characterization

Either RG or TG and B (MBP_RG_ and MBP_TG_, respectively) were mixed before use in a ratio 2 (RG or TG) to 1 (B). MBP_RG_ and MBP_TG_ had similar values of pH and TTA, being *ca*. 6.3 and 9 ml NaOH 0.1 M, respectively (Table 2). However, the concentration of lactic acid was significantly higher in MBP_RG_. Acetic acid was not detectable in any of the sample prior the fermentation. Significant differences were also found for TFAA and peptides concentrations, being higher in MBP_RG_ (Table 2). As expected, after 24 h of fermentation with *L*. *plantarum* T6B10 and *W*. *confusa* BAN8, the value of pH of FMBP_RG_ and FMBP_TG_ was lower compared to the corresponding unfermented doughs (MBP_RG_ and MBP_TG_, respectively), with lower value in FMBP_TG_. On the contrary, values of TTA increased during fermentation, being significantly higher in FMBP_TG_ compared to FMBP_RG_. Lactic acid concentration in FMBP_RG_ and FMBP_TG_ was *ca*. 10–100 times higher than MBP_RG_ and MBP_TG_, respectively (Table 2). Similar trend was found for acetic acid. Moreover, FMBP_RG_ and FMBP_TG_ contained different concentrations of both lactic and acetic acids, being higher in FMBP_TG_ and FMBP_RG_, respectively. QF was determined only in the fermented samples, being *ca*. 4.7. Fermented samples (FMBP_RG_ and FMBP_TG_) had significantly higher concentrations of TFAA (up to 80%) and peptides (up to 21%) compared to MBP_RG_ and MBP_TG_. Moreover, the presence of raw germ in the mixture (MBP_RG_ and FMBP_RG_) led to higher values compared to heat-treated germ containing samples (MBP_TG_ and FMBP_TG_). As regard to the nutritional properties, MBP_RG_ contained higher contents of phytic acids as compared to MBP_TG_, however, any significant differences were found in term of total phenols. Moreover, the radical scavenging activity in the WSE, was lower in MBP_RG_. The fermentation led to a decrease and increase of the phytic acid concentration (up to 50%) and radical scavenging activity in the WSE (up to 30 times), respectively (Table 2). A slight increase of the concentration of total phenols was also found (Table 2). The minimum concentration of the crude enzyme extract that failed to give a detectable zone of hydrolysis was 35 ± 2.7 μg/ml for MBP_RG_.

**Table 2:** Biochemical and nutritional properties of maize milling by-products doughs: MBP_RG_, unfermented mixture of raw germ and bran; MBP_TG_, unfermented mixture of heat-treated germ and bran; FMBP_RG_ fermented mixture of raw germ and bran; FMBP_TG_, fermented mixture of heat-treated germ and bran.

	MBP_RG_	MBP_TG_	FMBP_RG_	FMBP_TG_
**Biochemical characteristics**				
pH	6.41 ± 0.4^c^	6.23 ± 0.5^c^	4.21 ± 0.02^b^	4.04 ± 0.01^a^
TTA (ml NaOH 0.1 M)	8.4 ± 0.6^a^	9.6 ± 0.5^b^	31.2 ± 0.5^c^	35.5 ± 0.3^d^
Lactic acid (mmol/Kg)	5.22 ± 0.2^b^	0.70 ± 0.4^a^	60.47 ± 0.6^c^	66.48 ± 0.4^d^
Acetic acid (mmol/Kg)	n.d.	n.d.	7.0 ± 0.6^b^	4.67 ± 0.4^a^
QF	n.d.	n.d.	4.0 ± 0.2^b^	5.4 ± 0.3^a^
TFAA (mg/Kg)	1431 ± 12^b^	816 ± 15^a^	1905 ± 14^d^	1470 ± 20^c^
Peptide concentration (mg/g)	38.4 ± 0.4^b^	35.3 ± 0.4^a^	46.5 ± 0.6^d^	42.1 ± 0.4^c^
**Nutritional characteristics**				
Phytic acid (g/100 g)	1.10 ± 0.03^c^	0.81 ± 0.02^b^	0.53 ± 0.02^a^	0.55 ± 0.04^a^
Total phenols (mmol/Kg)	1.6 ± 0.4^a^	1.7 ± 0.2^a^	1.8 ± 0.4^a^	2.0 ± 0.3^a^
Radical scavenging activity (%) on ME	52.3 ± 0.3^b,c^	51.3 ± 0.7^b^	52.8 ± 0.4^c^	50.9 ± 0.3^a^
Radical scavenging activity (%) on WSE	1.4 ± 0.04^a^	5.8 ± 0.03^b^	42.5 ± 0.3^d^	41.4 ± 0.6^c^

*The data are the means of three independent experiments ± standard deviations (n = 3). ^a-d^Values in the same row with different superscript letters differ significantly (p < 0.05). WSE, Water/Salt extract; ME, Methanolic extract. Fermented milling by-products doughs (FMBP_RG_ and FMBP_TG_) (DY 200) were fermented with Lactobacillus plantarum T6B10 and Weissella confusa BAN8 at 30°C for 24 h.*

### Biochemical and Nutritional Characterization of Breads

Biochemical and nutritional characteristics of breads are summarized in Table 3. Similar values of moisture and *a*_w_ were found between breads (MBP_RG_-B, MBP_TG_-B, FMBP_RG_-B, and FMBP_TG_-B). No significant differences were found between enriched breads and WB (Table 3). As expected, the values of pH, TTA and concentrations of lactic and acetic acids (up to 6 times) were lower and higher, respectively, in breads enriched with fermented milling by-products (FMBP_RG_-B and FMBP_TG_-B) as compared to MBP_RG_-B, MBP_TG_-B, and WB. Moreover, MBP_RG_-B was characterized by higher value of pH and concentrations of lactic and acetic acids as compared to MBP_TG_-B, however, the TTA did not differ significantly. Any significant differences were found in term of acetic acids between in FMBP_RG_-B and FMBP_TG_-B. According to these results, the QF was higher in fermented milling by-products containing breads. Significant higher (up to *ca*. 3 times) concentrations of TFAA were found in enriched breads as compared to WB. Among experimental breads, FMBP_RG_-B and FMBP_TG_-B contained higher (58–91%, respectively) concentration of TFAA as compared to the corresponding MBP_RG_-B and MBP_TG_-B, however, MBP_RG_-B had higher contents than MBP_TG_-B. Similar trend was found for peptides contents. Lowest content was found for WB. Higher values were found in enriched breads (24–70%) as compared to WB, however, the use of fermented milling by-products led to a higher content of peptides (up to *ca*. 30%) as compared to the corresponding un-fermented milling by-products breads. Overall, the use of RG corresponded to higher values, as compared to TG (Table 3).

**Table 3:** Biochemical and nutritional properties of breads: MBP_RG_-B, containing unfermented mixture of raw germ and bran (MBP_RG_,12.5%, wt/wt); MBP_TG_-B, containing unfermented mixture of heat-treated germ and bran (MBP_TG_, 12.5%, wt/wt); FMBP_RG_-B, containing fermented mixture of raw germ and bran (FMBP_RG_, 25% wt/wt); FMBP_TG_-B, containing fermented mixture of heat-treated germ and bran (FMBP_TG_, 25% wt/wt); WB, wheat flour bread.

	MBP_RG_-B	MBP_TG_-B	FMBP_RG_-B	FMBP_TG_-B	WB
**Biochemical characteristics**					
Moisture (%)	32.9 ± 0.3^b^	31.9 ± 0.6^a^	32.5 ± 0.8^b^	31.6 ± 0.7^a,b^	31.0 ± 0.2^a^
*a*_w_	0.93 ± 0.05^a^	0.94 ± 0.06^a^	0.94 ± 0.04^a^	0.94 ± 0.06^a^	0.92 ± 0.02^a^
pH	5.59 ± 0.05^c^	5.46 ± 0.06^b^	4.22 ± 0.04^a^	4.17 ± 0.01^a^	5.61 ± 0.3^c^
TTA (ml NaOH 0.1 M)	8.2 ± 0.6^a^	8.2 ± 0.4^a^	15.4 ± 0.4^b^	16.6 ± 0.3^c^	9.1 ± 0.3^a^
Lactic acid (mmol/Kg)	1.53 ± 0.02^b^	0.76 ± 0.04^a^	25.1 ± 0.6^c^	29.2 ± 0.4^d^	3.3 ± 0.5^a^
Acetic acid (mmol/Kg)	3.93 ± 0.05^b^	0.83 ± 0.03^a^	5.7 ± 0.5^c^	5.3 ± 0.6^c^	1.27 ± 0.3^a^
QF	0.4 ± 0.1^a^	0.9 ± 0.2^b^	4.4 ± 0.2^c^	5.5 ± 0.3^d^	2.6^a^
TFAA (mg/Kg)	214 ± 10^b^	142 ± 11^a^	338 ± 12^d^	272 ± 15^c^	134 ± 10^a^
Peptide concentration (mg/g)	33.1 ± 0.5^b^	30.2 ± 0.4^a^	41.2 ± 0.5^d^	38.4 ± 0.4^c^	24.2 ± 0.1
**Nutritional properties**					
Protein (%)	13.0 ± 0.2^b^	13.1 ± 0.4^b^	12.9 ± 0.6^b^	13.1 ± 0.3^b^	6.3 ± 0.1^a^
Fat (%)	5.9 ± 0.5^b^	6.5 ± 0.3^b^	5.9 ± 0.3^b^	6.5 ± 0.5^b^	0.61 ± 0.04^a^
Available carbohydrates (%)	70.2 ± 1.7^b^	69.4 ± 1.3^b^	69.4 ± 1.3^b^	69.5 ± 0.4^b^	76.5 ± 0.9^a^
Total dietary fibers (%)	10.9 ± 0.6^b^	10.8 ± 0.5^b^	10.8 ± 0.7^b^	10.8 ± 0.3^b^	1.87 ± 0.02^a^
*IVPD* (%)	48 ± 3^b^	44 ± 1^b^	72 ± 1^c^	70 ± 2^c^	39 ± 1^a^
*HI* (%)	93 ± 3^c^	87 ± 1^c^	82 ± 2^b^	77 ± 1^a^	100 ± 1
Phytic acid (mg/100 g)	340 ± 4^d^	270 ± 5^c^	70 ± 4^a^	140 ± 2^b^	234 ± 6^e^
Total phenols (mmol/Kg)	0.82 ± 0.04^a^	0.99 ± 0.03^b^	1.07 ± 0.04^c^	1.20 ± 0.02^d^	2.39 ± 0.03^e^
Radical scavenging (%) on ME	39.4 ± 0.5^b^	53.1 ± 0.4^d^	49.8 ± 0.4^c^	58.0 ± 0.4^e^	20.3 ± 0.3^a^
Radical scavenging (%) on WSE	16.6 ± 0.3^a^	22.4 ± 0.5^c^	31.5 ± 0.5^d^	33.7 ± 0.4^e^	18.2 ± 0.3^b^

*The data are the means of three independent experiments ± standard deviations (n = 3). Data of protein, fat, carbohydrates and total fiber are expressed on dry weight. ^a-e^Values in the same row with different superscript letters differ significantly (p < 0.05). WSE, Water/Salt extract; ME, Methanolic extract. Fermented milling by-products doughs (FMBP_RG_ and FMBP_TG_) (DY 200) were fermented with Lactobacillus plantarum T6B10 and Weissella confusa BAN8 at 30°C for 24 h. Doughs for breadmaking had DY 180.*

The higher concentrations of TFAA and peptides in breads containing the fermented milling by-products reflected on the IVPD which was up to 70% with respect to MBP_TG_ and MBP_RG_ (*ca*. 40%). WB had the lowest IVPD, 13–23% and 79–84% higher values were found for MBP_RG_-B and MBP_TG_-B and FMBP_RG_-B and FMBP_TG_-B, respectively. Compared to WB, MBP_RG_-B and MBP_TG_-B and FMBP_RG_-B and FMBP_TG_-B were characterized by significant lower values of the HI. Moreover, up to 13% lower values of the HI were observed in FMBP_RG_-B and FMBP_TG_-B as compared to the corresponding un-fermented milling by-products enriched breads. The lowest value was found for FMBP_TG_-B (*ca*. 77%). The use of milling by-products as ingredient in breadmaking led to high fiber (up to *ca*. 11% of d.m) and protein of *ca*. (13% of d.m) contents in all breads as compared to WB, regardless the fermentation process and the germ heat-treatment.

According to the results of MBP, phytic acid content was lower in MBP_TG_-B compared to MBP_RG_-B. Lower values were found when FMBP_RG_ and FMBP_TG_ were included in bread formula (Table 3). Nevertheless, higher values of phytic acids were found in MBP_TG_-B and MBP_RG_-B compared to WB (Table 3). The presence of milling by-products in bread led to higher concentration of total phenols than WB. Values 10–34% and 45–62% higher were found in MBP_RG_-B and MBP_TG_-B and FMBP_RG_-B and FMBP_TG_-B, respectively, as compared WB. MBP_TG_-B was characterized by higher concentration of total phenols as well as higher radical scavenging in both ME and WSE as compared to MBP_RG_-B. When FMBP_TG_ and FMBP_RG_ were used to fortify the bread, higher values of radical scavenging activities (ME and WSE) and total phenols concentration were found as compared to the corresponding un-fermented samples (Table 3).

### Structural Properties and Sensory Profile of the Breads

After baking, structural and sensory analysis were carried out on MBP_RG_-B, MBP_TG_-B, FMBP_RG_-B, FMBP_TG_-B, and WB. The specific volume of the breads was influenced by the addition of milling by-products and fermentation process indeed the inclusion of milling by-products caused a decrease of the specific volume up to *ca*. 6% (MBP_RG_-B, MBP_TG_-B) as compared to WB. However, FMBP_RG_-B, FMBP_TG_-B showed higher specific volume than the corresponding MBP_RG_-B and MBP_TG_-B, respectively. Nevertheless, WB was characterized by the highest value. On the contrary, the hardness seemed to be influenced by both heat-treatment (increase) and fermentation (decrease) (Table 4). Compared to WB, the inclusion of milling by-products led to an increase of the hardness, however, it was lower in fermented products compared to un-fermented. Moreover, the presence of RG led to an increase of the value as compared to TG.

**Table 4:** Structural properties of breads: MBP_RG_-B, containing unfermented mixture of raw germ and bran (MBP_RG_,12.5%, wt/wt); MBP_TG_-B, containing unfermented mixture of heat-treated germ and bran (MBP_TG_, 12.5%, wt/wt); FMBP_RG_-B, containing fermented mixture of raw germ and bran (FMBP_RG_, 25% wt/wt); FMBP_TG_-B, containing fermented mixture of heat-treated germ and bran (FMBP_TG_, 25% wt/wt); WB, wheat flour bread.

	MBP_RG_-B	MBP_TG_-B	FMBP_RG_-B	FMBP_TG_-B	WB
Specific volume (cm^3^/g)	2.27 ± 0.4^c^	2.29 ± 0.3^c^	2.80 ± 0.2^b^	2.70 ± 0.2^b^	2.91 ± 0.02^a^
Resilience	0.79 ± 0.02^a^	0.89 ± 0.04^b^	0.82 ± 0.03^a^	0.86 ± 0.03^a,b^	0.85 ± 0.04^a,b^
Cohesiveness	0.47 ± 0.05^a^	0.48 ± 0.02^a^	0.49 ± 0.04^a^	0.49 ± 0.02^a^	0.70 ± 0.07^b^
Gumminess (N)	24.5 ± 0.6^c^	17.1 ± 0.4^b^	38.2 ± 0.7^d^	25.1 ± 0.8^c^	7.3 ± 0.2^a^
Chewiness (g)	1930 ± 21^c^	1530 ± 13^b^	3140 ± 26^e^	2150 ± 17^d^	825 ± 13^a^
Hardness (g)	7720 ± 47^d^	5070 ± 35^c^	5150 ± 49^c^	3590 ± 56^b^	2890 ± 22^a^
**Color crust**					
*L*	46.3 ± 1.9^b^	42.3 ± 1.2^a^	47.3 ± 2.7^b^	42.6 ± 1.8^a^	68.1 ± 0.7^c^
*a*	3.2 ± 0.3^b^	5.01 ± 1.2^c^	3.6 ± 0.4^b^	5.9 ± 0.4^c^	2.5 ± 0.1^a^
*b*	17.4 ± 0.6^a^	17.3 ± 0.4^a^	18.7 ± 0.7^b^	18.2 ± 0.5^a,b^	23.4 ± 0.3^b^
Δ*E*	48.5 ± 1.2^b^	52.8 ± 0.7^c^	47.9 ± 0.6^b^	52.8 ± 0.6^c^	33.1 ± 0.5^a^

*The data are the means of three independent experiments ± standard deviations (n = 3) ^a-e^Values in the same row with different superscript letters differ significantly (p < 0.05). Fermented milling by-products doughs (FMBP_RG_ and FMBP_TG_) (DY 200) were fermented with Lactobacillus plantarum T6B10 and Weissella confusa BAN8 at 30°C for 24 h. Doughs for breadmaking had DY 180.*

**FIGURE 2:**
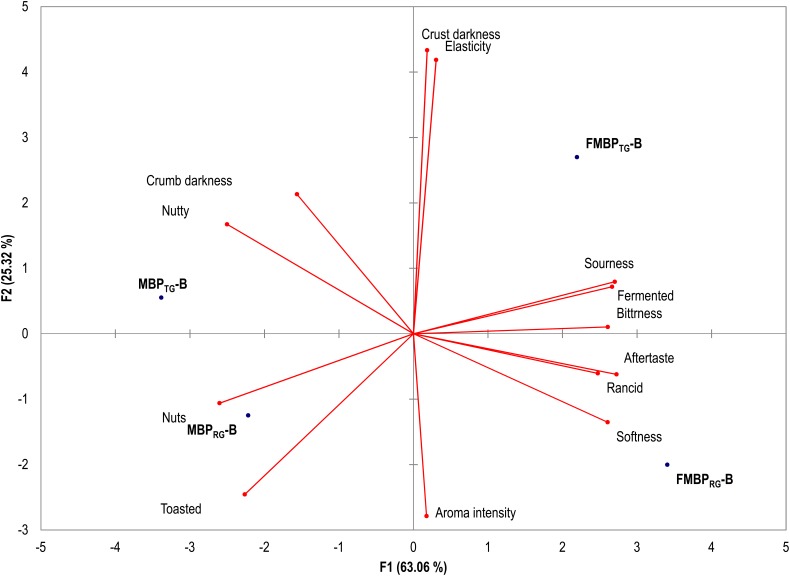
Principal Components Analysis (PCA) based on the sensory attributes of breads: MBP_RG_-B, containing unfermented mixture of raw germ and bran (MBP_RG_,12.5%, wt/wt)_;_ MBP_TG_-B, containing unfermented mixture of heat-treated germ and bran (MBP_TG_, 12.5%, wt/wt); FMBP_RG_-B, containing fermented mixture of raw germ and bran (FMBP_RG_, 25% wt/wt); FMBP_TG_-B, containing fermented mixture of heat-treated germ and bran (FMBP_TG_, 25% wt/wt). Fermented milling by-products doughs (FMBP_RG_ and FMBP_TG_) (DY 200) were fermented with *Lactobacillus plantarum* T6B10 and *Weissella confusa* BAN8 at 30°C for 24 h. Doughs for breadmaking had DY 180.

Aiming at highlighting the effect of the fermentation on the sensory profile of fiber-rich breads, MBP_RG_-B, MBP_TG_-B, FMBP_RG_-B, and FMBP_TG_-B were subjected to sensory analysis. The results are shown in Figure 2. PCA, representing 88% of the total variance of the data, showed that FMBP_TG_-B and FMBP_RG_-B were both scattered on the right zone of the plane because sharing the fermented, sourness, softness and bitterness aroma and taste. On the left part of the plane, the MBP_TG_-B, MBP_RG_-B were distributed. The perception of rancidity, although with very low scores, was found in MBP_RG_-B and FMBP_RG_-B. The analysis also pointed up perfect separation between breads containing RG and TG. Attributes such as toasted, nuts and nutty were perceived, as demonstrated by PCA, only in MBP_TG_-B and FMBP_TG_-B.

## Discussion

The growing interest of consumers in balanced nutrition, due to the increase in the number of overweight people in Western society, has refocused the food industry on the merits of including some form of dietary fiber in food products (Redgwell and Fischer, 2005). Besides the well-known functional effects related to the nutrient absorption modification and the prebiotic capability, the importance of the dietary fiber is increasing due to its beneficial effects on the reduction of cholesterol levels and the risk of colon cancer (Lattimer and Haub, 2010). Health authorities, world-wide, recommend an increase of cereal derived foods in diet, since recognized as potential source of dietary fiber, especially when employed as whole grains (Dahl et al., 2015). The widespread consumption of cereals all over the world and the tradition of breadmaking give bread an important position in international nutrition being a popular staple food for ages. Nevertheless, white bread, obtained by refined flour, is the most consumed type of bread. Therefore, aiming at meeting the recommendations regarding the dietary fiber intake, the development of high fiber bread could be a promising strategy. Producing fortified/enriched products without compromising their sensory appeal, seems to be a real challenge for the food industry. Indeed, the consumer wants variety, good taste but no constraints and sometime placed nutrition second in importance to taste in factors for food selection (Sandvik et al., 2018). Among ingredients, milling by-products (bran and germ) are considered suitable fiber sources to be used as bread ingredients (Hemdane et al., 2016). Nevertheless, the increase in fiber content causes several issues, such as the decrease of loaf volume and softness, and the decrease in consumer acceptability (Wang et al., 2002). Moreover, in the case of germ, a stabilization prior the inclusion in flour is strictly required to avoid oxidative rancidity (Leenhardt et al., 2006), due to the concomitant presence of lipids and lipases (Tovey and Hobsley, 2004). Maize and its milling by-products are often contaminated with mycotoxins produced by fungi of the genera *Aspergillus*, *Fusarium*, and *Penicillium* (Fountain et al., 2014; Munkvold et al., 2019). Management of mycotoxin problems requires a multifaceted approach including preharvest and postharvest strategies (Okeke et al., 2015; Burns et al., 2018) for preventing mycotoxin accumulation, and a strict control of the matrix intended for food and feed uses (Munkvold et al., 2019). From the nutritional point of view, besides the positive contribution of tocopherols, fiber, high quality protein, iron, zinc and vitamins (Naves et al., 2009), the abundance of anti-nutritional factors (e.g., phytic acid) characterizing milling by-products may lower the overall quality of fortified breads (Rizzello et al., 2012; Hemdane et al., 2016).

The application of the sourdough-type fermentation, involving LAB as natural or selected starters, has largely been proposed as valuable biotechnology able to solve the above mentioned technological, stabilization and nutritional problems related to the employment of cereal milling by-products as bread ingredient (Arte et al., 2015; Pontonio et al., 2017), allowing the exploitation of by-products generally treated as wastes. The production of value-added products from food processing wastes have gained worldwide attention (Wang and Chen, 2010; Burns et al., 2018) due to the economical but also environmental implications (Galanakis, 2012).

Among cereals, maize (Pranjal et al., 2017) is grown throughout the world and although the good nutritional profile its use as food ingredient is second to the fuel production (Ranum et al., 2014). Similarly, to wheat, maize milling methods produce a variety of economically and nutritionally valuable co-products, which can be used as food ingredient (Naves et al., 2009).

This study aimed at improving the nutritional profile of wheat bread using raw- and heat-treated wastes of maize milling process fermented by selected LAB.

Apart from the moisture, RG and TG had similar proximate composition. As expected, cell density of the different microbial groups considered was in TG very low, as the consequence of the thermal process, while in RG and B samples microbial contaminations were observed, at different level. However, *Enterobacteriaceae* were not found (<10 cfu/1 g) showing the good hygienic status of all the samples (Cordier, 2006) meeting food quality and safety standards. Either RG or TG were combined, with B in a ratio of 2:1 according to their presence in the maize kernel (*ca*. 11 and 5%, respectively) (Gwirtz and Garcia-Casal, 2013), before use and characterization. Doughs were prepared (MBP_RG_ and MBP_TG_) mixing milling by-products and water in a ratio of 1:1 and fermented (FMBP_RG_ and FMBP_TG_) with two LAB strains, *L*. *plantarum* T6B10 and *W*. *confusa* BAN8, previously selected according to their pro-technological, biochemical and nutritional features (kinetics of growth and acidification, proteolytic, phytase and antioxidant activity) (Pontonio et al., 2015; Rizzello et al., 2016). Overall, MBP_TG_ was characterized by lower concentration of lactic acid, TFFA, peptide and phytic acid as compared to MBP_RG_. The partial denaturation of the endogenous proteases and the significant decrease of the resident microbiota due to the heat-treatment might explain the lower values of TFFA and peptides concentrations. Similarly, the dejection of LAB, and more in general, microbial density led to a reduction of lactic acid in the TG.

Lactic acid bacteria fermentation led, as expected, to a relevant decrease of the pH due to the synthesis of lactic and acetic acids (Gobbetti et al., 2005). Moreover, FMBP_RG_ and FMBP_TG_ were characterized by higher concentration of TFAA compared to MBP_RG_ and MBP_TG_. Sourdough fermentation resulted in an increase of amino acid concentrations due to the proteolytic activity of sourdough LAB and endogenous proteases which have been activated under the acidic conditions of sourdough fermentation (Thiele et al., 2002). TFAA and peptides concentrations were higher in FMBP_RG_ than in FMBP_TG,_ thus hypothesizing a contribution of the activity of endogenous proteases of the raw germ to the microbial proteolysis. The lower lactic acid concentration detected in FMBP_RG_ may be explained by the reduced acidification efficiency of the inoculated LAB, affected by the competition of the endogenous microbiota.

The digestibility of protein, bioavailability of amino acids and protein quality of foods can be weakened by the presence of dietary anti-nutritional factors (Soetan and Oyewole, 2009). Phytic acid is an anti-nutritional factor because works as an excellent chelator of minerals, complexes the basic amino acid group of proteins, thus decreasing their dietary bioavailability (Febles et al., 2002). Fermentation with LAB contributed to create the optimal environment for phytase (myo-inositol-hexakisphosphate phosphohydrolase, EC 3.1.3.8) (Poutanen et al., 2009) which decreased the concentration of phytic acid, down to trace in FMBP_RG_. The optimal pH of a purified phytase from maize seedlings was 4.8 (Laboure et al., 1993). The proteolysis operated by endogenous proteases and microbial peptidase during the fermentation may have led to the release of peptides with antioxidant activity, thus explaining the increase of the radical scavenging activity in the WSE in FMBP_RG_ and FMBP_TG_. Although the LAB fermentation often leads to an increase of the solubilization of phenolic compounds having antioxidant activity, due to the biological acidification and the microbial enzymatic activity (e.g., feruloyl-esterase and β-glucosidase activities) radical scavenging activity as well as in the ME and total phenols concentration FMBP_RG_ and FMBP_TG_ were not significantly different than MBP_RG_ and MBP_TG_.

**FIGURE 3:**
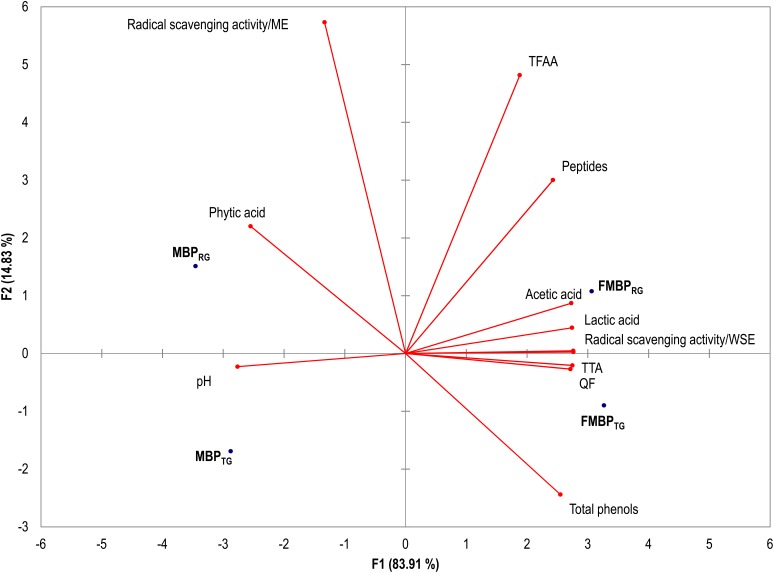
Principal Components Analysis (PCA) based on the biochemical and nutritional properties of the milling by-products doughs: MBP_RG_, unfermented mixture of raw germ and bran_;_ MBP_TG_, unfermented mixture of heat-treated germ and bran; FMBP_RG_ fermented mixture of raw germ and bran; FMBP_TG_, fermented mixture of heat-treated germ and bran. Fermented milling by-products doughs (FMBP_RG_ and FMBP_TG_) (DY 200) were fermented with *Lactobacillus plantarum* T6B10 and *Weissella confusa* BAN8 at 30°C for 24 h.

Data of the biochemical and nutritional characterization of MBP_RG_, MBP_TG_, FMBP_RG_, and FMBP_TG_ were subjected to Principal Components Analysis (PCA) (Figure 3). The factors explained the 98.7% of the total variance and clearly differentiated the MBP_RG_ and MBP_TG_ form the corresponding fermented FMBP_RG_ and FMBP_TG_. Indeed, the former and the latter are scattered on the left and right part of the plane, respectively. Overall, FMBP_RG_ and FMBP_TG_ seem to be characterized by a more complex profile than the corresponding MBP_TG_ and MBP_RG_, with improved nutritional features. According to the literature, maize germ contains lipases which are responsible for the fatty acid oxidation leading to unstable product with poor quality (Paradiso et al., 2008; Rizzello et al., 2010). Under this study conditions, the lactic acid fermentation was proposed as valuable alternative to heat treatment aiming at decreasing the lipase activity of maize germ. Indeed, MBP_RG_ was the only sample showing activity, however, a strong inhibition was achieved through the fermentation (FMBP_RG_). As expected, similar results were observed when the heat treatment (MBP_TG_ and FMBP_TG_) was used; however, it may have decrease of the nutritional value of maize germ (Paradiso et al., 2008; Rizzello et al., 2010).

FMBP_RG_ and FMBP_TG_ were used as ingredient for the manufacture of wheat bread, whose characteristics were compared to those of breads manufactured with MBP_RG_ and MBP_TG_. Overall, the nutritional properties of white bread have been improved through the inclusion of milling by-products in the formula. However, the best improvements were observed when FMBP_RG_ and FMBP_TG_ were used. Indeed, according to the high content of organic acids in fermented samples, FMBP_RG_-B and FMBP_TG_-B were characterized by optimal QF for sensory profile (Minervini et al., 2012) and acetic acid concentration, associated to the extended microbiological shelf-life (Katina, 2005). Improvements in TFAA and peptides concentrations, as well as the phytic acid degradation and the enhancement of the radical scavenging activity (WSE) in bread containing fermented milling by-products, were also found. According to the literature, when dietary fiber is used in breadmaking, the loaf volume and shelf-life are often compromised. Bran is the main responsible for the weak structure and poor baking quality (low bread volume and elasticity of crumb) in fiber enriched bread (Katina, 2005). These effects on the dough structure are due to the dilution of the gluten network, which in turn impairs gas retention rather than gas production. Thus, the supplementation of dietary fiber requires changes in processing techniques to produce baked goods with good consumer quality. The sourdough was proposed as strategy to overcome the downsides in breadmaking due to the high fiber contents (Katina, 2005; Pontonio et al., 2017). Under these study conditions, the specific volume and the hardness of FMBP_TG_-B and FMBP_RG_-B were higher and lower, respectively, than the corresponding MBP_TG_-B and MBP_RG_-B corroborating the previous findings (Katina, 2005; Katina et al., 2006b; Rizzello et al., 2012).

High fiber content (Regulation EC No. 1924/2006, 2006) was achieved including milling by-products in bread, however, the fermentation with LAB positively affected other nutritional features, i.e., IVPD and HI, leading to a bread with high nutritional profile. High protein content and digestibility was achieved when fermented milling by-products were used to fortify wheat bread. A high content of protein (*ca*. 13% of d.m.) as well as values of IVPD up to *ca*. 60% were observed. The latter were mainly due to the intense proteolysis operated by endogenous and microbial enzymes (Pontonio et al., 2017). The predicted GI was also investigated, through the study of the starch hydrolysis kinetic *in vitro* conditions. Due to the biological acidification, lactic acid bacteria fermentation can be used to decrease the starch hydrolysis during digestion (De Angelis et al., 2009). Indeed, FMBP_RG_-B and FMBP_TG_-B showed lower values as compared to the corresponding (MBP_RG_-B and MBP_TG_-B).

The sensory acceptability of the breads was assessed by a panel test, and although all breads were appreciated, those containing fermented milling by-products showed a more balanced profile. Moreover, breads fortified with FMBP_RG_ and FMBP_TG_ were characterized by acidic aroma and taste typical of sourdough breads (Katina et al., 2006a).

The results of this study demonstrate that fermentation with LAB led the improvement of nutritional properties of maize milling by-products through the increase of free amino acids, peptides concentrations and antioxidant activity, and the decrease of the antinutritional phytic acid. Fermentation also caused the chemical stabilization of the by-products through the inhibition of the lipase activity, thus suggesting an alternative use to heat-treatment which often impair the nutritional profile of germ.

## Conclusion

This study demonstrates the potential of fermentation to convert maize bran and germ, commonly considered food wastes, into nutritive improvers, meeting nutritional and sensory requests of modern consumer. The fortification of wheat bread with maize milling by-products allows the increase in dietary fiber and proteins compared to a conventional wheat bread. When fermented, milling by-products also conferred to fortified bread the advantages commonly related to the sourdough fermentation, such as the increase of the protein digestibility, the decrease of the starch hydrolysis, the degradation of phytic acid. Moreover, LAB fermentation as pre-processing treatment positively affects the textural properties and the sensory profile of the breads.

## Author Contributions

EP conceived the study, elaborated the results, and wrote the draft of the manuscript. CD carried out the experiments. MG was the scientific advisor and responsible for financial contribution. CR supervised the work and critically revised the manuscript. All authors read and approved the final manuscript.

## Conflict of Interest Statement

The authors declare that the research was conducted in the absence of any commercial or financial relationships that could be construed as a potential conflict of interest.

## References

[B1] Akeson W. R. Stahmann M. A. (1964). A pepsin pancreatin digest index of protein quality evaluation. *J. Nutr.* 83 257–261. 10.1093/jn/83.3.257 14191426

[B2] American Association of Cereal Chemists [AACC] (2010). *International Approved Methods of Analysis.* St Paul: AACC International.

[B3] Arte E. Rizzello C. G. Verni M. Nordlund E. Katina K. Coda R. (2015). Impact of Enzymatic and microbial bioprocessing on protein modification and nutritional properties of wheat bran. *J. Agr. Food Chem.* 63 8685–8693. 10.1021/acs.jafc.5b03495 26365885

[B4] Bradford M. M. (1976). A rapid and sensitive method for the quantitation of microgram quantities of protein utilizing the principle of protein-dye binding. *Anal. Biochem.* 72 248–254. 10.1016/0003-2697(76)90527-3 942051

[B5] Burns P. Borgo M. F. Binetti A. Puntillo M. Bergamini C. Páez R. (2018). Isolation, characterization and performance of autochthonous spray dried lactic acid bacteria in maize micro and bucket-silos. *Front. Microbiol.* 9:2861. 10.3389/fmicb.2018.02861 30555432 PMC6282064

[B6] Capriles V. D. Areas J. A. G. (2013). Effects of prebiotic inulin-type fructans on structure, quality, sensory acceptance and glycemic response of gluten-free breads. *Food Funct.* 4 04–10. 10.1039/c2fo10283h 23032642

[B7] Church F. C. Swaisgood H. E. Porter D. H. Catignani G. L. (1983). Spectrophotometric assay using o-phthaldialdehyde for determination of proteolysis in milk and isolated milk proteins. *J. Dairy Sci.* 66 1219–1227. 10.3168/jds.S0022-0302(83)81926-2

[B8] Cordier J. L. (2006). “Enterobacteriaceae,” in *Emerging Foodborne Pathogens*, eds Motarjemi Y. Adams M. (Cambridge: Woodhead Publishing Limited), 450–475. 10.1533/9781845691394.2.450

[B9] Dahl W. J. Gainesville F. L. Stewart M. L. Honolulu H. I. (2015). Position of the academy of nutrition and dietetics: health implications of dietary fiber. *J. Acad. Nutr. Diet.* 115 1861–1870. 10.1016/j.jand.2015.09.003 26514720

[B10] De Angelis M. Damiano N. Rizzello C. G. Cassone A. Di Cagno R. Gobbetti M. (2009). Sourdough fermentation as a tool for the manufacture of low-glycemic index white wheat bread enriched in dietary fibre. *Eur. Food Res. Technol.* 229 593–601. 10.1007/s00217-009-1085-1

[B11] Febles C. I. Arias A. Hardisson A. Rodrıìguez-Alvarez C. Sierra A. (2002). Phytic acid level in wheat flours. *J. Cereal Sci.* 36 19–23. 10.1006/jcrs.2001.0441

[B12] Fernstrand A. M. Bury D. Garssen J. Verster J. C. (2017). Dietary intake of fibers: differential effects in men and women on perceived general health and immune functioning. *Food Nutr. Res.* 61:1297053. 10.1080/16546628.2017.1297053 28469542 PMC5404421

[B13] Fountain J. Scully B. Ni X. Kemerait R. Lee D. Zhi-Yuan C. (2014). Environmental influences on maize-*Aspergillus flavus* interactions and aflatoxin production. *Front. Microbiol.* 5:40. 10.3389/fmicb.2014.00040 24550905 PMC3913990

[B14] Frauenlob J. Scharl M. D’Amico S. Schoenlechner R. (2018). Effect of different lipases on bread staling in comparison with Diacetyl tartaric ester of monoglycerides (DATEM). *Cereal Chem.* 95 367–372. 10.1002/cche.10047

[B15] Galanakis C. M. (2012). Recovery of high added-value components from food wastes: conventional, emerging technologies and commercialized applications. *LWT Food Sci. Tech.* 26 68–87. 10.1016/j.tifs.2012.03.003

[B16] Georgsson F. (2015). Guidelines for sensory evaluation of bread. *NMKL Proced.* 31 1–25.

[B17] Gobbetti M. De Angelis M. Corsetti A. Di Cagno R. (2005). Biochemistry and physiology of sourdough lactic acid bacteria. *Trends Food Sci. Technol.* 16 57–69. 10.1016/j.tifs.2004.02.013

[B18] Gwirtz J. A. Garcia-Casal M. N. (2013). Processing maize flour and corn meal food products. *Ann. N.Y. Acad. Sci.* 1312:75. 10.1111/nyas.12299 24329576 PMC4260129

[B19] Haglund A. Johansson L. Dahlstedt L. (1998). Sensory evaluation of wholemeal bread from ecologically and conventionally grown wheat. *J. Cereal Sci.* 27 199–207. 10.1006/jcrs.1997.0155

[B20] Hemdane S. Jacobs P. J. Dornez E. Verspreet J. Delcour J. A. Courtin C. M. (2016). Wheat (*Triticum aestivum L*.) bran in bread making. *Compr. Rev. Food Sci. Food Safety* 15 28–42. 10.1111/1541-4337.12176 33371577

[B21] Howarth N. C. Saltzman E. Roberts S. B. (2001). Dietary fiber and weight regulation. *Nutr. Rev.* 59 129–139. 10.1111/j.1753-4887.2001.tb07001.x 11396693

[B22] Kaczmarczyk M. M. Miller M. J. Freund G. G. (2012). The health benefits of dietary fiber: beyond the usual suspects of type 2 diabetes mellitus, cardiovascular disease and colon cancer. *Metab. Clin. Exp.* 61 1058–1066. 10.1016/j.metabol.2012.01.017 22401879 PMC3399949

[B23] Katina K. (2005). *Sourdough: a Tool for the Improved Flavour, Texture and Shelf-Life of Wheat Bread.* Doctoral dissertation, University of Helsinki, Helsinki.

[B24] Katina K. Juvonen R. Laitila A. Flander L. Nordlund E. Kariluoto S. (2012). Fermented wheat bran as a functional ingredient in baking. *Cereal Chem.* 89 126–134. 10.1094/CCHEM-08-11-0106

[B25] Katina K. Heinio R. L. Autio K. Poutanen K. (2006a). Optimization of sourdough process for improved sensory profile and texture of wheat bread. *LWT Food Sci. Tech.* 39 1189–1202. 10.1016/j.lwt.2005.08.001

[B26] Katina K. Salmenkallio-Marttila M. Partanen R. Forssell P. Autio K. (2006b). Effects of sourdough and enzymes on staling of high-fibre wheat bread. *LWT Food Sci. Tech.* 39 479–491. 10.1016/j.lwt.2005.03.013

[B27] Laboure A. M. Gagnon J. Lescure A. M. (1993). Purification and characterization of a phytase (myo-inositol-hexakisphosphate phosphohydrolase) accumulated in maize (*Zea mays*) seedlings during germination. *Biochem. J.* 295 413–419. 10.1042/bj2950413 8240238 PMC1134897

[B28] Lattimer J. M. Haub M. D. (2010). Effects of dietary fiber and its components on metabolic health. *Nutrients* 2 1266–1289. 10.3390/nu2121266 22254008 PMC3257631

[B29] Lawrence R. C. Fryer T. F. Reiter B. (1967). Rapid method for the quantitative estimation of microbial lipases. *Nature* 213 1264–1265. 10.1038/2131264a0

[B30] Leenhardt F. Lyan B. Rock E. Boussard A. Potus J. Chanliaud E. (2006). Genetic variability of carotenoid concentration, and lipoxygenase and peroxidase activities among cultivated wheat species and bread wheat varieties. *Eur. J. Agron.* 25 170–176. 10.1016/j.eja.2006.04.010

[B31] Lin Y. H. Wimer L. T. Huang A. H. (1983). Lipase in the lipid bodies of corn scutella during seedling growth. *Plant Pathol.* 73 460–463. 10.1104/pp.73.2.460 16663239 PMC1066484

[B32] Lopez H. W. Adam A. Leenhardt F. Scalbert A. Remesy C. (2001). Control of the nutritional value of bread. *Industries des Ce.* 124 15–20.

[B33] Mamhoud A. Nionelli L. Bouzaine T. Hamdi M. Gobbetti M. Rizzello C. G. (2016). Selection of lactic acid bacteria isolated from tunisian cereals and exploitation of the use as starters for sourdough fermentation. *Int. J. Food Microbiol.* 225 9–19. 10.1016/j.ijfoodmicro.2016.03.004 26974248

[B34] Minervini F. Di Cagno R. Lattanzi A. De Angelis M. Antonielli L. Cardinali G. (2012). Lactic acid bacterium and yeast microbiotas of 19 sourdoughs used for traditional/typical Italian breads: interactions between ingredients and microbial species diversity. *Appl. Environ. Microbiol.* 78 1251–1264. 10.1128/AEM.07721-11 22156414 PMC3273004

[B35] Munkvold G. P. Arias S. Taschl I. Gruber-Dorninger C. (2019). “Mycotoxins in corn: occurrence, impacts, and management,” in *Corn Chemistry and Technology*, ed. Serna S. O.-Saldivar (Cambridge: Woodhead Publishing), 235–287. 10.1016/B978-0-12-811971-6.00009-7

[B36] Naves M. M. De Castro M. V. L. De Mendonça A. L. Santos G. G. Silva M. S. (2009). Corn germ with pericarp in relation to whole corn: nutrient contents, food and protein efficiency, and protein digestibility-corrected amino acid score. *Ciênc. Tecnol. Aliment.* 31 264–269. 10.1590/S0101-20612011000100040

[B37] Nionelli L. Currib N. Curiel J. A. Di Cagno R. Pontonio E. Cavoski I. (2014). Exploitation of Albanian wheat cultivars: characterization of the flours and lactic acid bacteria microbiota, and selection of starters for sourdough fermentation. *Food Microbiol.* 44 96–107. 10.1016/j.fm.2014.05.011 25084651

[B38] Nionelli L. Montemurro M. Pontonio E. Verni M. Gobbetti M. Rizzello C. G. (2018a). Pro-technological and functional characterization of lactic acid bacteria to be used as starters for hemp (*Cannabis sativa L*.) sourdough fermentation and wheat bread fortification. *Int. J. Food Microbiol.* 279 14–25. 10.1016/j.ijfoodmicro.2018.04.036 29715603

[B39] Nionelli L. Pontonio E. Gobbetti M. Rizzello C. G. (2018b). Use of hop extract as antifungal ingredient for bread making and selection of autochthonous resistant starters for sourdough fermentation. *Int. J. Food Microbiol.* 266 173–182. 10.1016/j.ijfoodmicro.2017.12.002 29223035

[B40] Noort M. W. J. Van Haaster D. Hemery Y. Schols H. A. Hamer R. J. (2010). The effect of particle size of wheat bran fractions on bread quality - evidence for fibre - protein interactions. *J. Cereal Sci.* 52 59–64. 10.1016/j.jcs.2010.03.003

[B41] Okeke C. A. Ezekiel C. N. Nwangburuka C. C. Sulyok M. Ezeamagu C. O. Adeleke R. A. (2015). Bacterial diversity and mycotoxin reduction during maize fermentation (steeping) for Ogi production. *Front. Microbiol.* 6:1402. 10.3389/fmicb.2015.01402 26697001 PMC4678208

[B42] Osborne T. B. (1907). *Proteins of the Wheat Kernel.* Washington, DC: Carnegie Institution, 1–119. 10.5962/bhl.title.26152

[B43] Paradiso V. M. Summo C. Trani A. Caponio F. (2008). An effort to improve the shelf life of breakfast cereals using natural mixed tocopherols. *J. Cereal Sci.* 47 322–330. 10.1016/j.jcs.2007.04.009

[B44] Pontonio E. Lorusso A. Gobbetti M. Rizzello C. G. (2017). Use of fermented milling by-products as functional ingredient to develop a low-glycaemic index bread. *J. Cereal Sci.* 77 235–242. 10.1016/j.jcs.2017.08.022

[B45] Pontonio E. Nionelli L. Curiel J. A. Sadeghi A. Di Cagno R. Gobbetti M. (2015). Iranian wheat flours from rural and industrial mills: exploitation of the chemical and technology features, and selection of autochthonous sourdough starters for making breads. *Food Microbiol.* 47 99–110. 10.1016/j.fm.2014.10.011 25583343

[B46] Poutanen K. Flander L. Katina K. (2009). Sourdough and cereal fermentation in a nutritional perspective. *Food Microbiol.* 26 693–699. 10.1016/j.fm.2009.07.011 19747602

[B47] Pranjal Y. Alok A. Reeva S. Ishwar S. Tanushri K. Arunava P. (2017). Advances in maize transformation technologies and development of transgenic maize. *Front. Plant Sci.* 7:1949. 10.3389/fpls.2016.01949 28111576 PMC5216042

[B48] Preedy V. R. Watson R. R. Patel V. B. (2011). *Flour and Breads and their Fortification in Health and Disease Prevention.* London: Academic Press.

[B49] Ranum P. Peña-Rosas J. P. Garcia-Casal M. N. (2014). Global maize production, utilization, and consumption. *Ann. N. Y. Acad. Sci.* 1312 105–112. 10.1111/nyas.12396 24650320

[B50] Redgwell R. J. Fischer M. (2005). Dietary fiber as a versatile food component: an industrial perspective. *Mol. Nutr. Food. Res.* 49 421–535. 10.1002/mnfr.200500028 15926144

[B51] Regulation EC No. 1924/2006 (2006). *Regulation EC No. 1924/2006 the European. (Parliament) and of the Council of 20 December 2006 on Nutrition and Health Claims Made on Foods.* Available at: https://eur-lex.europa.eu/eli/reg/2006/1924/2012-11-29

[B52] Rizzello C. G. Coda R. Mazzacane F. Minervini D. Gobbetti M. (2012). Micronized by-products from debranned durum wheat and sourdough fermentation enhanced the nutritional, textural and sensory features of bread. *Food Res. Int.* 46 304–313. 10.1016/j.foodres.2011.12.024

[B53] Rizzello C. G. Curiel J. Nionelli L. Vincentini O. Di Cagno R. Silano M. (2014). Use of fungal proteases and selected sourdough lactic acid bacteria for making wheat bread with an intermediate content of gluten. *Food Microbiol.* 37 59–68. 10.1016/j.fm.2013.06.017 24230474

[B54] Rizzello C. G. Lorusso A. Montemurro M. Gobbetti M. (2016). Use of sourdough made with quinoa (Chenopodium quinoa) four and autochthonous selected lactic acid bacteria for enhancing the nutritional, textural and sensory features of white bread. *Food Microbiol.* 56 1–13. 10.1016/j.fm.2015.11.018O 26919812

[B55] Rizzello C. G. Nionelli L. Coda R. De Angelis M. Gobbetti M. (2010). Effect of sourdough fermentation on stabilization, and chemical and nutritional characteristics of wheat germ. *Food Chem.* 119 1079–1089. 10.1016/j.foodchem.2009.08.016

[B56] Rosell C. M. Bajerska J. El Sheikha A. F. (2016). *Bread and Its Fortification Bread Nutrition and Health Benefits.* Didcot: Tyalor, and Francis Group.

[B57] Sandvik P. Nydahl M. Kihlberg I. Marklinder I. (2018). Consumers’ health-related perceptions of bread - implications for labeling and health communication. *Appetite* 121 285–293. 10.1016/j.appet.2017.11.092 29154885

[B58] Singh K. P. Mishra A. Mishra H. N. (2012). Fuzzy analysis of sensory attributes of bread prepared from millet-based composite flours. *LWT Food Sci. Tech.* 48 276–282. 10.1016/j.lwt.2012.03.026

[B59] Sivam A. S. Sun-Waterhouse D. Quek S. Conrad O. (2010). Properties of bread dough with added fiber polysaccharides and phenolic antioxidants: a review. *J. Food Sci.* 75:8. 10.1111/j.1750-3841.2010.01815.x 21535512 PMC3032915

[B60] Sjovall O. Virtalaine T. Lapvetelainen A. Kallio H. (2000). Development of rancidity in wheat germ analyzed by headspace gas chromatography and sensory analysis. *J. Agric. Food Chem.* 48 3522–3527. 10.1021/jf981309t 10956143

[B61] Slinkard K. Singleton V. (1977). Total phenol analysis: automation and comparison with manual methods. *Am. J. Enol. Viticult.* 28 49–55. 10.3390/molecules15128618 21116230 PMC6259195

[B62] Soetan K. O. Oyewole O. E. (2009). The need for adequate processing to reduce the anti-nutritional factors in plants used as human foods and animal feeds. *Afr. J. Food Sci.* 3 223–232.

[B63] Stephen A. M. Champ M. M.-J. Cloran S. J. Fleith M. Van Lieshout L. Mejborn H. (2017). Dietary fibre in Europe: current state of knowledge on definitions, sources, recommendations, intakes and relationships to health. *Nutr. Res. Rev.* 1:42. 10.1017/S095442241700004X 28676135

[B64] Thiele C. Gänzle M. G. Vogel R. F. (2002). Contribution of sourdough lactobacilli, yeast, and cereal enzymes to the generation of amino acids in dough relevant for bread flavor. *Cereal Chem.* 79 45–45. 10.1094/CCHEM.2002.79.1.45

[B65] Tovey F. I. Hobsley M. (2004). Milling of wheat, maize and rice: effects on fibre and lipid content and health. *World J. Gastroenterol.* 10 1695–1696. 10.3748/wjg.v10.i12.1695 15188488 PMC4572251

[B66] Wang J. Rosell C. M. De Barber C. B. (2002). Effect of the addition of different fibres on wheat dough performance and bread quality. *Food Chem.* 79 221–226. 10.1016/S0308-8146(02)00135-8

[B67] Wang S. Y. Chen C. T. (2010). Effect of allyl isothiocyanate on antioxidant enzyme activities, flavonoids and post-harvest fruit quality of blueberries (*Vaccinium corymbosum L*., cv. Duke). *Food Chem.* 122 1153–1158. 10.1016/j.foodchem.2010.03.106

[B68] Weiss W. Vogelmeier C. Gorg A. (1993). Electrophoretic characterization of wheat grain allergens from different cultivars involved in bakers’ asthma. *Electrophoresis* 14 805–816. 10.1002/elps.11501401126 8404825

[B69] WHO, (2018). *Obesity and Overweight.* Available at: https://www.who.int/news-room/fact-sheets/detail/obesity-and-overweight

[B70] Zwietering M. H. Jongeberger I. Roumbouts F. M. Van’t Riet K. (1990). Modelling of bacterial growth curve. *Appl. Environ. Microbiol.* 56 1875–1881. 16348228 10.1128/aem.56.6.1875-1881.1990 PMC184525

