# Supplementary material for: Maize Milling By-Products: From Food Wastes to Functional Ingredients Through Lactic Acid Bacteria Fermentation
Source: Front Microbiol. 2019 Mar 19;10:561. doi: 10.3389/fmicb.2019.00561 (PMC6434969; doi:10.3389/fmicb.2019.00561)
Supplement: Supplementary file 1 [file Table_1.pdf]

Supplementary Table S1. List of the lactic acid bacteria strains used in this study. Source of isolation and references are also reported.

Strain	Species	Source of Isolation	References
SAN9	*Leuc. citreum*	Wheat sourdough	Pontonio et al., 2015
SAN5			
NEY6	*W. confusa*		
SAN7			
NEY26			
KAS3			
BAN8			
BAN50			
Ney5			
Ney1			
STF 14	*L. plantarum*		Nionelli et al., 2014
STF 15			
DAJ 9			
DAJ 13			
DAJ 15			
DAJ 27			
LVS 15			
LIN 2			
LIN 3			
LIN 23			
LVS 26	*Lc. lactis*		
STF 1	*Leuc. citreum*		
STF 2			
STF 18			
DAJ 5			
LIN 21			
PRO 6			
PRO 7			
PRO 15			
LVS 5	*Leuc. mesenteroides*		
LVS 21			
LIN 8			
LIN 12			
LIN 22			
PRO 21			
STF 22	*P. pentosaceus*		
S1N2	*L.curvatus*		

MK2			Mamhoud et al., 2016
MK4			
MA2			
S3N2	*L. farciminis*		
OBJ1	*L. nantensis*		
TBJ4			
O2B2	*P.acidilactici*		
T1B2			
O1A1			
BTBJ1			
BDMB1			
BTMB3			
BTMB1			
BDT1			
BDBJ3			
BDK1			
BDMB4			
BTS1			
BTMB2			
T0A16	*L. rossiae*	Raw and spontaneously fermented quinoa flour	Rizzello et al., 2016
T1A14	*L. plantarum*		
T1B6			
T6A10			
T6B4			
T6C5			
T0A2			
T0B3			
T6B10			
T6C20			
T6C16			
T1C14			
T0C3			
T0A6			
T0A10			
T1B16			
T1A13	*P. pentosaeus*		
T1B11			
H10	*E. feacium*	Hop	Nionelli et al., 2018a
H41			
H51			
H46	*L. brevis*		

H35	*L. helvetcus*		
H1	*L. plantarum*		
H48			
H34	*P. acidilactici*		
H3	*P. pentosaceus*		
H11			
11M9	*L. plantarum*	Hemp flour	Nionelli et al., 2018b
12M9			
15M9			
16M9			
12MM1	*Leuc. mesenteroides subsp mesenteroides*		
10MM1	*P. acidilactici*		
9MM1			
6MM0			
19S1A	*P. pentosaceus*		
19S1B			
19S1D			
LB2	*L. plantarum*	Wheat germ	Rizzello et al., 2010
LB4			
G7	*W. confusa*		
G9			
G2	*P. pentosaceus*		

L., *Lactobacillus*; *Lc.*, *Lactococcus*; Leuc., *Leuconostoc*; *P.*, *Pediococcus*; *W.*, *Weissella*;
